# Utilizing passive sensing data to provide personalized psychological care in low-resource settings

**DOI:** 10.12688/gatesopenres.13117.2

**Published:** 2021-03-02

**Authors:** Prabin Byanjankar, Anubhuti Poudyal, Brandon A Kohrt, Sujen Man Maharjan, Ashley Hagaman, Alastair van Heerden

**Affiliations:** 1 Transcultural Psychosocial Organization (TPO) Nepal, Kathmandu, 44600, Nepal; 2 Division of Global Mental Health, Department of Psychiatry and Behavioral Sciences, George Washington School of Medicine and Health Sciences, Washington, DC, 20036, USA; 3 Department of Social and Behavioral Sciences, Yale School of Public Health, Yale University, New Haven, CT, 06510, USA; 4 Center for Methods in Implementation and Prevention Science, Yale School of Public Health, Yale University, New Haven, CT, 06510, USA; 5 Centre for Community Based Research, Human and Social Capabilities, Human Sciences Research Council, Pietermaritzburg, South Africa; 6 Medical Research Council/Wits Developmental Pathways for Health Research Unit, Department of Pediatrics, Faculty of Health Sciences, University of the Witwatersrand, Johannesburg, South Africa

**Keywords:** Passive sensing data, mobile health, low resource settings, behavioral disorders, mother-child interaction, postpartum depression, psychotherapy

## Abstract

**Background: **With the growing ubiquity of smartphones and wearable devices, there is an increased potential of collecting passive sensing data in mobile health. Passive data such as physical activity, Global Positioning System (GPS), interpersonal proximity, and audio recordings can provide valuable insight into the lives of individuals. In mental health, these insights can illuminate behavioral patterns, creating exciting opportunities for mental health service providers and their clients to support pattern recognition and problem identification outside of formal sessions. In the Sensing Technologies for Maternal Depression Treatment in Low Resource Settings (StandStrong) project, our aim was to build an mHealth application to facilitate the delivery of psychological treatments by lay counselors caring for adolescent mothers with depression in Nepal.

**Methods: **This paper describes the development of the StandStrong platform comprising the StandStrong Counselor application, and a cloud-based processing system, which can incorporate any tool that generates passive sensing data. We developed the StandStrong Counselor application that visualized passively collected GPS, proximity, and activity data. In the app, GPS data displays as heat maps, proximity data as charts showing the mother and child together or apart, and mothers’ activities as activity charts. Lay counselors can use the StandStrong application during counseling sessions to discuss mothers’ behavioral patterns and clinical progress over the course of a five-week counseling intervention. Achievement Awards based on collected data can also be automatically generated and sent to mothers. Additionally, messages can be sent from counselors to mother’s personal phones through the StandStrong platform.

**Discussion: **The StandStrong platform has the potential to improve the quality and effectiveness of psychological services delivered by non-specialists in diverse global settings.

## Introduction

New data collection techniques employing mobile technology are being explored worldwide to identify acceptable and safe ways of understanding human behavior that are non-invasive and do not interfere with daily activities. Data can be collected using mobile phone sensors such as accelerometers, digital cameras, microphones, Bluetooth sensors, and the Global Positioning System (GPS) ^1^. Many health applications have explored the potential of using passive sensing data to record human activity and movement ^2– 5^.

This type of information has the potential to transform mental health care. Unlike diabetes, hypertension, and cardiac dysrhythmias, which can now be monitored during daily life using portable devices, there have not been objective measures of mental health that can be remotely monitored by mental health care providers. Passive sensing data collection can change this because information on physical activity, interpersonal interactions, sleep, movement within one’s community, and other behavioral indicators of improving or worsening mental health status can be recorded. If this information can be unobtrusively and confidentially collected and shared with mental health workers, then treatment providers can tailor treatment to individual clients’ needs and contexts. For example, if a mother were being treated for post-partum depression and the therapy goals included spending more time with one’s infant or spending more time in social engagement and less time alone, then being able to monitor these behaviors could help a therapist identify when behavioral change was occurring or when barriers were encountered.

Having a window into a therapy client’s behaviors is especially helpful in low resource settings where the person delivering the therapy may not have a mental health professional background and where clients may have low literacy rates, impeding traditional pen-and-paper approaches to monitoring one’s behavioral change. There is a global push to increase the use of non-specialists to deliver psychological therapies—a process known as task-shifting or task-sharing mental health services—in order to reach the populations most in need in low resource settings around the world ^6– 9^.

It was against this backdrop of increased technological potential to use passive sensing on mobile devices and growing efforts to expand mental health services delivered by non-specialists that we developed the Sensing Technologies for Maternal Depression Treatment in Low Resource Settings (StandStrong) application for use with android smartphones. The concept of StandStrong is that applications on a depressed mother’s smartphone can capture four types of information: a) the auditory environment, to measure social interactions through speech; b) movement, as a measure of physical activity versus sedentary behavior; c) GPS data, to assess movement throughout the community; and d) mother-infant proximity, as captured with a passive Bluetooth beacon. 

We piloted the StandStrong platform in Nepal with a psychological treatment designed for use by non-specialists such as lay counselors or midwives. This intervention, the Healthy Activity Program (HAP) ^10^, is a psychological treatment delivered by non-specialists to treat depressed patients. It is based on the psychological principle of behavioral action, which focuses on changing behaviors to reduce avoidance and inactivity in order to improve thoughts and feelings ^11^. HAP was developed for depression treatment in India and has been successfully implemented in Nepal ^9, 11^. Recently, the Ministry of Health in Nepal has adopted HAP to be delivered by low-level health workers without prior training in mental health ^12^. The pilot implementation of StandStrong was for HAP delivered to adolescent and young mothers with postpartum depression ^12^.

This paper describes the implementation of this software with source code available on GitHub at https://github.com/mmhss (see *Software availability*).

## Methods

The StandStrong platform development occurred in the context of a study conducted in Chitwan, a southern district of Nepal, between May 2018 and October 2019. The implementation was tailored to psychological treatment for depressed adolescent and young mothers (ages 15–25 years) with infants under 12 months of age. The study protocol for implementation and evaluation of StandStrong has been published ^12^.

### Implementation

**Figure 1.**
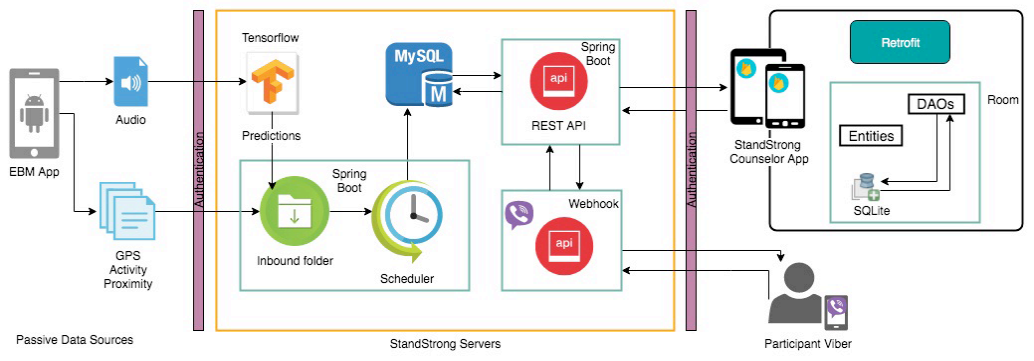
Architecture of StandStrong platform. EBM: Electronic Behavior Monitoring; DAO: Data Access Object.

Prior to the study, we developed an approach to capture passive sensing data that could be used by StandStrong platform. However, many freely available software tools, such as RADAR ^13^ and Passive Data Kit ^14^, can be used to capture passive data from smart devices. In our current study, the StandStrong platform was designed for use with android smartphones to process the data collected through a passive data collection tool, and then provide visualizations of the data for a lay counsellor delivering the psychological treatment. The platform comprises freely available components, namely, TensorFlow service (TF-Audio), Scheduler (StandStrong ELT), web services (StandStrong REST API), Viber Webhook, and the StandStrong Counsellor App. Figure 1 shows the StandStrong Architecture with server locations and data flow. Each tool’s source code and application hosted locations are summarized in the *Software availability* section and a full user guide is available as *Extended data* ^15^.

***Rationale for selected passive sensing domains.*** We have included four domains that can create a picture of the daily life of the mothers. Mother-child proximity, along with audio data can capture mother-child interaction. Audio data combined with GPS is intended to eventually be a proxy for social support. Activity data will capture the physical activity of the mother as a proxy for physical health. The combined information from these passive data can be used to create a picture of the daily schedules of mothers. This can be a proxy for routinization and stability. An instability of daily schedule as determined from the passive data can be a proxy for stress. Although we are moving towards a clearer understanding of how to analyze and operationalize to maximize the benefit of mothers, the initial idea with this study was just to surface behaviors of interest. We wanted to see if it was possible to create an app and integrate passive data within the app and whether such information would inform the counselor during the session. We were mostly concerned if passive data, in general, was a feasible form of data that we could collect in a rural setting. With what we have learned and the advances in the field mean we can move beyond the exploratory, particularly in operationalizing the domains, and what integrating passive data into these behavioral patterns means for mothers.

***Gathering passive sending data sources.*** Passive sensing data can be collected from smart devices such as smartphones and smartwatches through a variety of approaches. We had collected passive sensing data such as GPS, activity, proximity, and audio. Use of additional devices such as Bluetooth beacons along with smart devices can capture proximity data.

***Preparing passive sensing data.*** For audio data, mp3 recordings are fed to the TensorFlow service to generate audio predictions such as speech, music, vehicles, insects and so on. TensorFlow is an open software framework for machine learning ^16^, which we trained with YouTube human and environment sound models. The Amazon Web Services (AWS) S3 bucket is used for transferring files to the inbound folder in the cloud server. The scheduler job scans the incoming files periodically and loads them into the database. The web service in Heroku cloud provides the endpoints to access the data, which the StandStrong Counselor app gets for visualization. The platform also allows sending messages to and from a counselor and a mother using the popular Viber chat app. There is a wide range of approaches that could be used for analysis of the passive sensing data, and this is a rapidly evolving field. For the purposes of this initial work, our operationalization of passive sensing results was as follows:

- Proximity – Mother-child proximity was coded as hours when there was at least one reading of the mother and child in proximity. Counselors could use that information to prompt discussions with mothers about times of day when she was typically with or apart from her infant. The counselor and mother could jointly identify times when the mother wants to do activities or change behavior based on the proximity information, or identify potential changes in the patterns of spending time together or apart.
- GPS – The mother’s movement was coded based on coordinates of her movement, which could be used to produce heat maps (see Data visualization section below). The counselor could use this information to prompt discussions with mothers about her movements, with a focus on opportunities to pursue social support. There are many other ways in GPS could be analyzed that are not site-specific (e.g., radius of movement, farthest distance traveled, frequency of time outside the home); future uses of StandStrong could employ these analytic approaches in counseling.
- Activity – This was coded as ‘active’ if for any hour there was at least one period of physical activity (‘not-still’) identified by the accelerometer and “inactive” otherwise Counselors could use this information to encourage mothers to be physically active and target times when the mother could be more active.
- Audio – Audio was intended to be a proxy for social support by detecting times when there was human speech in the mother’s environment versus no speech detected. Data were coded so that every hour that had a least one 30-second recording with speech were reported as ‘speech’. Counselors could use this information to prompt discussions regarding when mothers were around others and/or speaking with their infant.

***Data visualization on the Counselor App.*** The StandStrong Counsellor App is an android-based mobile application that retrieves data through the web service and stores it on the device. While developing the system, one of the considerations was limited access to the internet; therefore, the app was designed using the ‘offline first’ principle.

**Figure 2.**
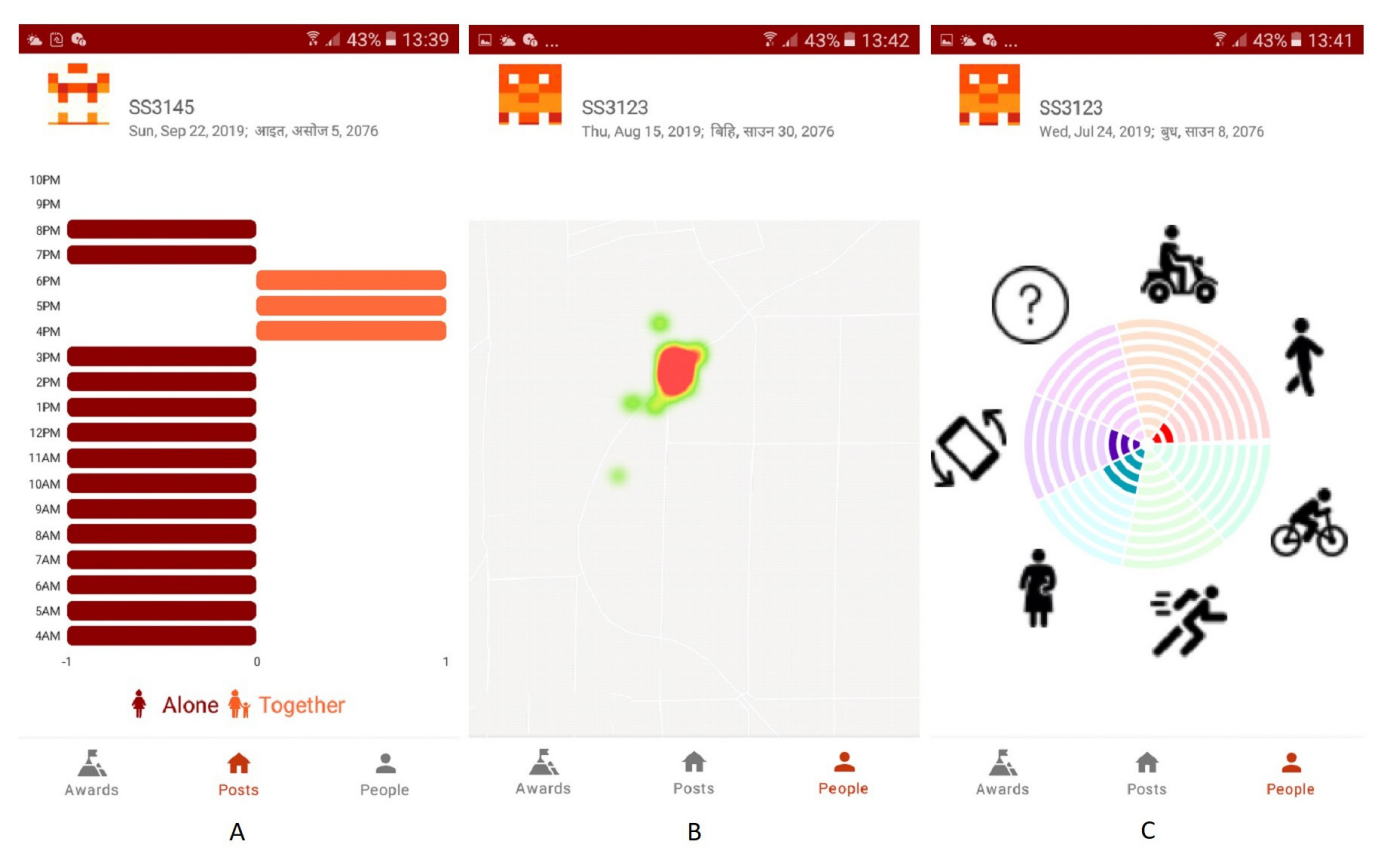
Proximity data ( **A**), Global Positioning System heat map ( **B**), and activity data ( **C**) as visualized in StandStrong app.

The StandStrong mobile app has the following features to provide visual representations of the passively collected data for counselor use:

1. A daily proximity bar chart displaying mother together with child, away from child and no data ( Figure 2).
2. A daily GPS heat map displaying mother’s movement ( Figure 2).
3. A daily activity chart displaying mother activities ( Figure 2).
4. A messaging feature that allows mothers to use Viber and send messages to the counselor’s StandStrong app.
5. A feature to record mothers’ weekly goals during the psychosocial counseling sessions.
6. Motivational award cards that deliver messages when behavioral goals and milestones are achieved.

The StandStrong platform components, web services (StandStrong REST API), StandStrong Counsellor App, and scheduler (StandStrong ELT) are available on Github for public access at https://github.com/mmhss (see *Software availability*).

### Operation

***StandStrong app in counselor’s tablets.*** Counselors can use the StandStrong app while providing counseling services to depressed adolescent mothers. The application’s use is both preparatory (the counselor explores the mother’s behavioral patterns to inform her session) and participatory (the counselor uses the visuals of the application with the mother to help discuss, identify, and set behavioral goals). We designed StandStrong for use with Samsung Galaxy Tab A7.0, which costs around $160 USD (purchased in Sept 2018) with accessories. The StandStrong app works on most android devices. However, we recommend a screen size of seven inches, RAM 1.5 GB, and Android 6.0 or higher. Figure 2 shows the proximity chart, GPS heat map, and activity chart for the participants. The proximity chart shows the time spent by the mother alone and with her child, the GPS heat map shows the locations where the mother spends her time, and the activity chart visualizes the mother’s activities, such as standing and running, as recorded by the mobile phone throughout the day. The detailed operation of the StandStrong Counselor App is documented and provided as *Extended data* ^15^.

***Equipment.*** Three types of equipment are used in the study. Different approaches and combinations of devices are available to collect passive sensing data including phones, smartwatches, Bluetooth beacons. For StandStrong, the counselors use tablets to access the passive sensing data visualized in the StandStrong app and discuss the data with mothers in counseling sessions. Detailed specifications of the equipment can be found in Table 1.

**Table 1.** Equipment used along with specifications.

Equipment name	Purpose	Specification
Smartphone	Smartphones with EBM app installed collects audio, activity, proximity, and GPS data. Mother carries the smartphone	Model: Samsung J2 Ace Cost: $114 Bluetooth version: 4.0 Android version: 6.0 Marshmallow
Tablet	Counselors use tablets to open the StandStrong app and discuss the data with the mothers during counseling sessions	Model: Samsung Galaxy Tab A7.0 Cost: $160 Bluetooth version: 4.0 Android version: 6.0 Marshmallow
Radbeacon Proximity Beacons	Bluetooth beacon on the child’s cloth transmits the signal to the EBM App installed in mother’s smartphone.	Model: RadBeacon Dot Cost: $10 Bluetooth Version: 4.0 Typical Line-of-sight range: 5m to 50m

EBM, Electronic Behavior Monitoring; GPS, Global Positioning System.

### Use cases

Below, we provide examples of input and output datasets, as well as two case studies to illuminate the platform’s architecture and pragmatic use. Use case 1 describes how StandStrong would be implemented from a project director perspective. Use case 2 describes how StandStrong is used by a lay counselor providing psychological treatment to a depressed adolescent mother.

***Implementation of passive sensing data collection.*** Our first step was to identify what types of information could be collected using passive sensing on mobile devices that would be technologically feasible and culturally acceptable in Nepal ^17^. Based on the feedback from users in prior studies, smartphones and Bluetooth beacons were considered the most appropriate tools for passive data collection in Nepal. In our prior study, we designed the Electronic Behavioral Monitoring (EBM) app, which can be installed on any android smartphone with Bluetooth version 4.0 to collect passive sensing data. EBM captures the four domains of data: audio recordings, physical activity, GPS location, and proximity of the phone to a passive Bluetooth beacon (attached to the infant’s clothing). 

To ensure affordability in a low-resource setting, we explored low cost devices that could run our applications. We chose the Samsung J2 Ace phone, which costs around $114. The EBM app collects audio data in m4a format; GPS location, activity and proximity of Bluetooth beacon are saved in CSV format on the device. During pilot testing, project staff retrieved the passive sensing data from the device once a week. Each week, around 200 MB of data are generated including seven GPS files, seven activity files, seven proximity files and 280 m4a files.

Through the EBM app, GPS, activity, proximity and audio data are passively collected at 15-minute intervals on the mother’s android smartphone and the information is stored locally on the smartphone as CSV, with the exception of audio files, which are m4a format. In EBM, the mobile phone’s accelerometer sensor and Google library are required to return movement information (walking, in vehicle, cycling, running, still) to crudely track activity. Proximity data are collected each time the mobile Bluetooth searches for the Bluetooth beacon attached to the child’s clothes. An audio clip of 30 seconds is collected every 15 minutes and saved to the phone. Finally, GPS location is recorded each time the mobile phone has some activity (screen on and off, phone calls and so on). When participants are enrolled, we provide a participant code so that generated data files are prefixed with the participant code, followed by the type of the data and date. The StandStrong platform is used by the counselor to visualize these data, as well as provide automated awards to the mother if she achieves behavioral targets.

Alternative passive data collections apps exist that could be used to collect the raw data for the StandStrong system. For example, the RADAR-base passive data collection app has the capability to capture data both on smartphones and smart watches ^13^. The app provides access to a wide variety of sensors including positioning sensors, movement sensors and social sensors supporting Bluetooth devices. Through a simple reformatting (e.g., using the StandStrong date format) collected data would be supported and usable by the StandStrong platform.

***Input dataset.*** During pilot testing, passive sensing data were collected by the EBM app, generating a separate file for each sensing data type. The naming convention for the file has several pieces of information each separated by a dash (-) and underscore (_). It starts with the mother identification code, followed by the delimiter “-” before the passive sensing data type name, followed by underscore and lastly, the date (e.g. SSXXXX-GPS_20190x0x.txt, SSXXXX-PROXIMITY_20190605.txt, SSXXXX-ACTIVITY_20190605.txt). The raw audio files captured by the EBM app are of around 30 seconds in length. On average, around 40 raw audio files (e.g. SSXXXX-EAR_201900x0x192732.m4a) are generated each day, which then are passed to the TensorFlow audio processor to generate the predictions. Tensorflow generates a CSV file (e.g. SSXXXX-AUDIO.csv) for weekly audio clips. The .csv file contains audio predictions that can be used for analysis. When using the RADAR-base passive data collection app as an alternative to the EBM app, the collected data for different sensors require transformation to make it compatible with the StandStrong platform. For example, the collected data should be exported as csv following the file naming convention used by StandStrong.

***Output dataset.*** The StandStong scheduler job loads the datasets into the “sstrong” database, which is implemented in the MySQL relational database system. The data transfer between the StandStrong components and database system is achieved by establishing a secured link. The first two database tables are configuration tables and named “project” and “mother”. The “project” table must have a setting for an inbound folder, the location where passive data files are uploaded in the system. There must be a record for the mother with a unique identification number, which is required to map the data loaded into the tables named “GPS”, “proximity”, “activity”, and “audio” for each mother. The database is the source providing daily information to the StandStrong Counselor App. The app gets the data through the web service, which is repeatedly synced to the database. During each attempt to sync, the app looks for new data added to the system. Our analytic approach has been to quantize down to the 15-minute level and then generate 24-hour mappings that include missing data so as to be able to compare behavioral rhythms day by day and across sensors which may not always be collected simultaneously. 

***Use case 1.*** A project director interested in implementing StandStrong in the context of psychological treatment would begin by preparing for software installation on tablets for counselors and on mobile phones for mothers. Access to the components in the architecture is needed, namely, the EBM app, StandStrong Counselor App, servers and AWS S3 Storage. The installation guide and user manual are provided as *Extended data* ^15^.

Psychosocial counselors can be trained to use the app. When procuring devices, low-cost Android smartphones like the Samsung J2 Ace phone are compatible to operate the StandStrong app. Thus, adding this passive sensing tool would be inexpensive from both technical and human resources perspectives. In terms of training, since it is passive data collection, mothers would not require training to use this technology. We will need to train the facilitators on using the technology, ensuring proper use during each visit, and integrating passive data in a psychosocial counseling session. Psychosocial counseling (HAP) is already a part of government-implemented training for psychosocial counselors in Nepal. Training of counselors in HAP in Nepal typically is delivered in approximately 5-days in the government curriculum. To integrate StandStrong, we recommend an extra day in the training. This is needed to train them on how to set up the passive sensing technology and troubleshooting. In addition, for each of the HAP sessions, the trainer should discuss with counselors what information is useful and can be incorporated into the session. Other training should include the use of awards and messaging with mothers. After training, counselors typically have weekly supervision with a HAP specialist, this will need to additionally include discussions of technological challenges and how the passive sensing information is being optimally used ^18^.

**Figure 3.**
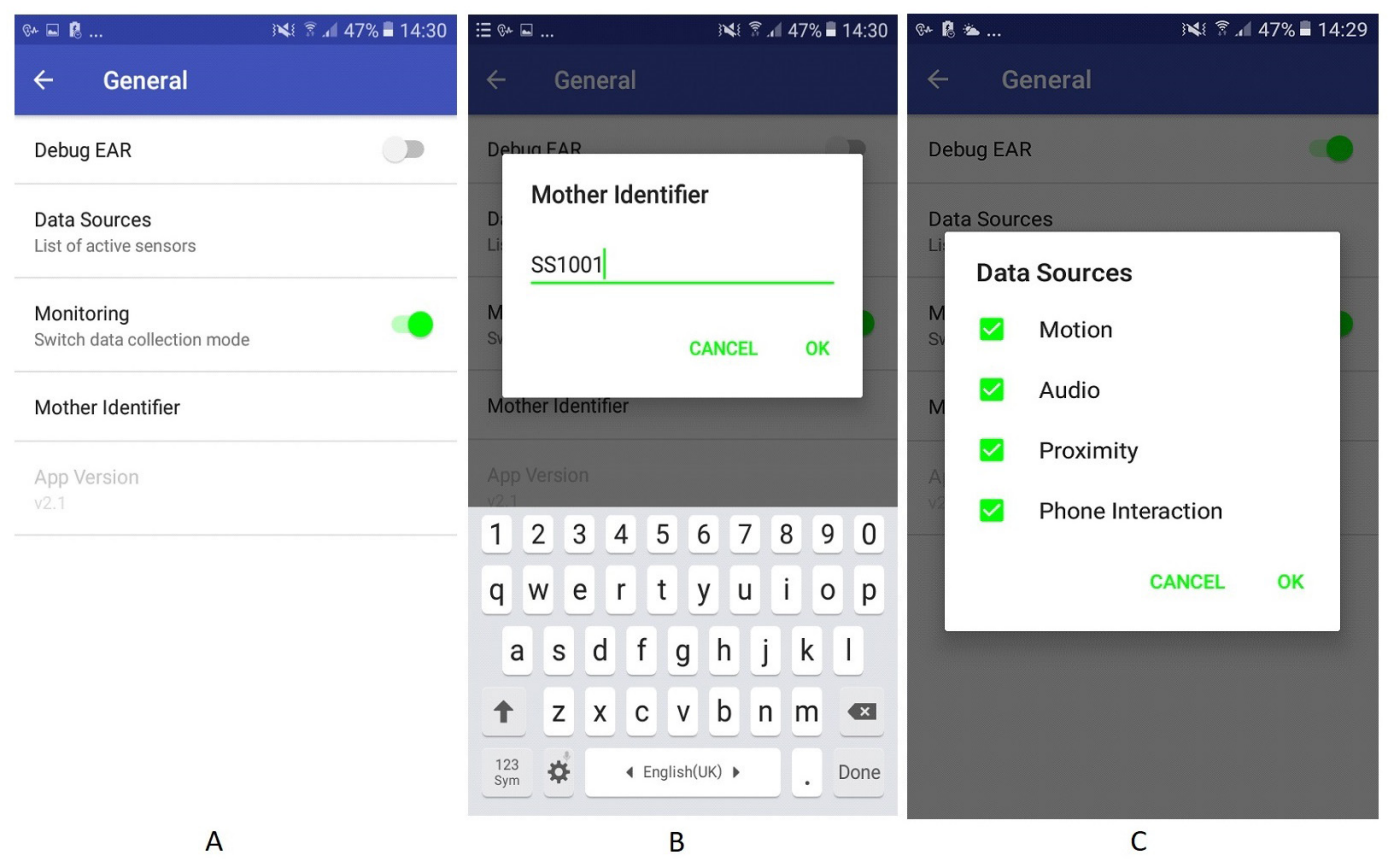
General tab ( **A**), mother identifier ( **B**), and passive data sources ( **C**) while setting up the Electronic Behavior Monitoring app.

When participants (e.g. mothers) are enrolled, they are all given a unique ID for data management in the StandStrong platform, e.g. SS-XXXX. Prior to enrollment, project staff visits the mother to sign the consent form. Two layers of consent were sought. First, we obtained a written consent from the mothers to use the devices. Second, we explained the technology to the family members and only proceeded with the study after the family members gave verbal consent. We addressed any privacy and confidentially-related concerns from the participants and their family members. In our study, it was integral to seek participant as well as family consent to ensure we addressed any privacy or confidentiality concerns of the family. We also shared a one-page description of the study with the participant and family, so she could describe the study to her family and friends, even when the study team was not present. Some of the key considerations related to privacy are, a) educating participants on how to delete the files from the phones, b) ongoing interactions with the participant and family throughout the study duration to ensure easy communication, and c) ensuring good rapport building with the participants so they feel comfortable to raise privacy concerns (if any) throughout the study period. In future studies, we will explore processing the audio files in the phone itself, so we do not have to store the audio clips on the phones. We have discussed the issues related to privacy and confidentiality in detail in a separate paper ^18^. Following participant and family consent, project staff visit the mother and give them a Samsung J2 Ace phone with the EBM. The EBM app is configured with the mother identification number, enabled audio recordings, access to device’s location and file system, and enabled motion, audio, proximity and phone interaction data sources ( Figure 3). The project staff ensure that GPS and mobile data are enabled for tracking GPS location. A Bluetooth beacon is attached to the infant’s clothes. RadBeacon Locate, a utility app, is also installed in the smartphone. In the case of RadBeacon Locate, the app is used to confirm that the signal from the proximity beacon is received by the phone. The RadBeacon app could be used at any time to see if the beacon is working or not. The mother’s information is then recorded in the database so that the passive data can be loaded into the system. This can be set up, either by inserting a new record directly through the SQL query or through the endpoints.

**Figure 4.**
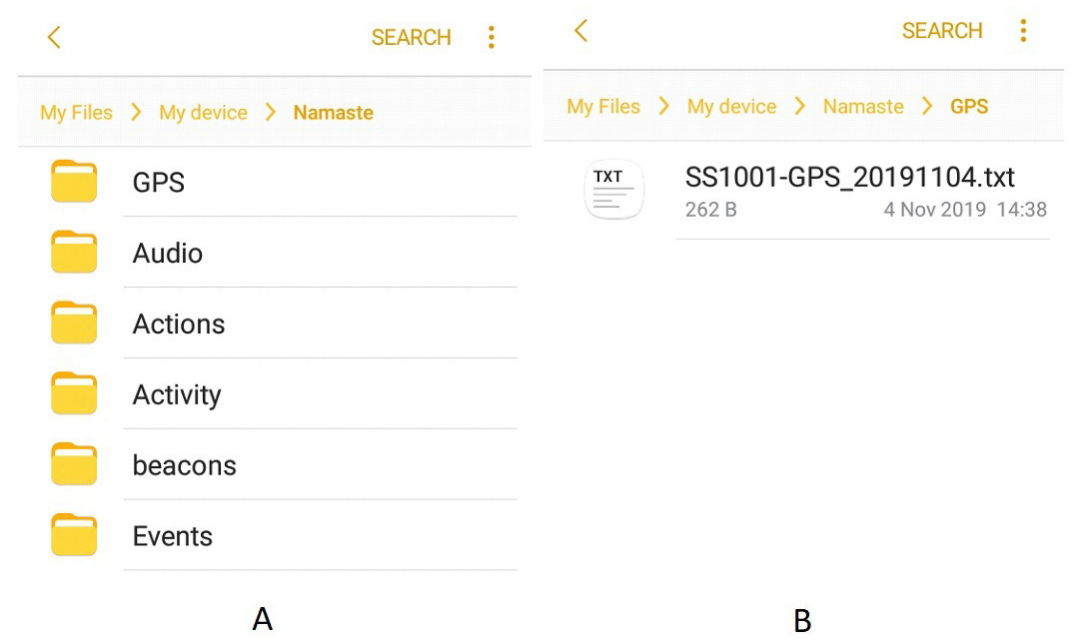
Folder structure ( **A**) and GPS file ( **B**) as captured by the Electronic Behavior Monitoring app. GPS, Global Positioning System.

At the end of a week of passive data collection, the project staff visit the mother’s home to download the passive data that are saved in a folder named Namaste in the mother’s phone ( Figure 4). In our current implementation, the data are collected weekly by the project staff. However, future implementation is intended to have realtime upload and processing so that mothers and counselors can immediately get feedback. The data are uploaded into a secured database with a week number identification for backup. Each of the passive data files are named with the mother’s identification number (without personally identifiable information). The project staff then upload the data text files into the AWS S3 bucket, from which they then are loaded into the server. Furthermore, the project staff confirm that the uploaded data files are successfully loaded into the database server using the utility tool every week.

Once the data is analyzed, it is visualized in the StandStrong app. Four types of data are analyzed to be used in the counseling sessions – a) Audio data, b) Proximity, c) Activity, and d) GPS data.

a. Audio – For the audio data, the audio recordings are not processed manually. The data file is directly uploaded to the TensorFlow app will compute the speech count percentage in the mother’s environment. An award was sent to the mother if there were presence of speech sounds during the day.
b. Proximity – Proximity chart shows hours per day that mother spent with her baby and hours spent apart.
c. Activity – Mothers’ activities are visualized in the app to show how much time she spends doing activities (running, walking, sitting etc.).
d. GPS – A heat map shows mothers’ geographical movement.

Using RADAR-base app would require setting up RADAR passive remote monitoring technology (pRMT) and the RADAR management portal. The portal allows adding a new participant to the study. Once a subject is created, the pRMT app can login to the management portal to provide permission for different sensors. The collected data then has to be exported to the format supported by the StandStrong Platform. More detail can be found at using pRMT is available on the RADAR-base website.

***Use case 2.*** Following Case 1, the next step is to integrate the passive data in the counselor’s StandStrong app. The four types of passive data collected using smartphones and Bluetooth beacons can be visualized in the StandStrong app installed on the counselor’s tablet. The counselor receives daily passive data updates for each mother. Before the weekly psychosocial session, the counselors synchronize the latest data in their tablet to discuss during the counseling session. Once downloaded, the data is available offline for sessions which are typically held in mothers’ homes that might not have reliable internet access. The counselor then meets the mother in her scheduled weekly psychosocial counseling session. During the counseling session, the counselor shares and discusses the mother’s data with her using the StandStrong app on her tablet. This information is integrated as a part of the psychosocial session, particularly for behavior activation, that reinforces positive behaviors among the mothers. For example, they may show the proximity chart, GPS heat map, and activity tracking while discussing mothers’ daily life and behavioral patterns in the last one week. Any weekly change in behavioral patterns between sessions can also be discussed. Finally, counselors can provide suggestions to the mothers on behavior change for better recovery based on her passive data. Besides interactions with the counselors during the psychosocial sessions, mothers can also send Viber text messages to counselors between sessions. These messages appear in the counselor’s StandStrong app on their tablet which they can respond through the app. Another reinforcing feature of StandStrong is “Awards” that are given to support certain self-care, daily routine, social interaction-related behavioral goals. This weekly progress is documented through passive data triggered behavioral change.

## Conclusion

Given the global burden of untreated depression and its intergenerational impacts on young mothers and their children, it is vital to find innovative approaches to scaling up psychological services. We have developed the StandStrong platform as a way to passively collect data on depressed mothers’ daily experience and then provide this information to non-specialist counselors who can personalize psychological treatment. The StandStrong platform has now been piloted and results will be forthcoming regarding the application of this approach in a real-world setting. These findings will inform the acceptability, feasibility, and mental health benefits of the StandStrong platform. With what we have learned in this study and the current development in the field of passive sensing technology, we can move beyond this exploratory stage. We are continually updating our app and strengthening our methodology, especially in the operationalization of the passive sensor indicators. Future studies will focus on defining indicators and validating passive sensing data constructs.

## Data availability

### Underlying data

All data underlying the results are available as part of the article and no additional source data are required.

### Extended data

Zenodo: Supplementary File - StandStrong Counselor App. https://doi.org/10.5281/zenodo.3709431 ^15^

Data are available under the terms of the Creative Commons Attribution 4.0 International license (CC-BY 4.0).

## Software availability

Source code of the components are available at GitHub organization link - https://github.com/mmhss. Source code of each component is available below.

StandStrong Counselor App

Source code available from: https://github.com/mmhss/sstrong-counselor

Archived source code at time of publication: https://doi.org/10.5281/zenodo.3709405 ^19^

Scheduler (StandStrong ELT)

Source code available from: https://github.com/mmhss/sstrong-import

Archived source code at time of publication: https://doi.org/10.5281/zenodo.3933064 ^20^

Messaging Webhook

Source code available from: https://github.com/mmhss/sstrong-webhook

Archived source code at time of publication: https://doi.org/10.5281/zenodo.3933074 ^21^

Web Service (StandStrong REST API)

Source code available from: https://github.com/mmhss/sstrong-rest-server

Archived source code at time of publication: https://doi.org/10.5281/zenodo.3933119 ^22^

License: GNU Affero General Public License v3 (AGPL-3.0)

## References

[1] Piwek L Ellis DA Andrews S: The Rise of Consumer Health Wearables: Promises and Barriers. *PLoS Med.* 2016;13(2):e1001953. 10.1371/journal.pmed.1001953 26836780 PMC4737495

[2] Galambos C Skubic M Wang S: Management of Dementia and Depression Utilizing In- Home Passive Sensor Data. *Gerontechnology.* 2013;11(3):457–468. 10.4017/gt.2013.11.3.004.00 24049513 PMC3773874

[3] Heintzman ND: A Digital Ecosystem of Diabetes Data and Technology: Services, Systems, and Tools Enabled by Wearables, Sensors, and Apps. *J Diabetes Sci Technol.* 2015;10(1): 35–41. 10.1177/1932296815622453 26685994 PMC4738231

[4] Torous J Onnela JP Keshavan M: New dimensions and new tools to realize the potential of RDoC: digital phenotyping via smartphones and connected devices. *Transl Psychiatry.* 2017;7(3):e1053. 10.1038/tp.2017.25 28267146 PMC5416670

[5] Gu WZY Zhou Z Liu X: SugarMate: non-intrusive blood glucose monitoring with smartphones. *Proc ACM Interact Mob Wearable Ubiquitous Technol.* 2017;1(3):1–27. 10.1145/3130919

[6] Patel V: The future of psychiatry in low- and middle-income countries. *Psychol Med.* 2009;39(11):1759–62. 10.1017/s0033291709005224 20162837

[7] Patel V Goel DS Desai R: Scaling up services for mental and neurological disorders in low-resource settings. *Int Health.* 2009;1(1):37–44. 10.1016/j.inhe.2009.02.002 21637318 PMC3081098

[8] Sikander S Ahmad I Atif N: Delivering the Thinking Healthy Programme for perinatal depression through volunteer peers: a cluster randomised controlled trial in Pakistan. *Lancet Psychiatry.* 2019;6(2): 128–139. 10.1016/S2215-0366(18)30467-X 30686386

[9] Jordans MJD Luitel NP Garman E: Effectiveness of psychological treatments for depression and alcohol use disorder delivered by community-based counsellors: two pragmatic randomised controlled trials within primary healthcare in Nepal. *Br J Psychiatry.* 2019;215(2):485–493. 10.1192/bjp.2018.300 30678744 PMC6878117

[10] Luitel NP Jordans MJD Kohrt BA: Treatment gap and barriers for mental health care: A cross-sectional community survey in Nepal. *PLoS One.* 2017;12(8):e0183223. 10.1371/journal.pone.0183223 28817734 PMC5560728

[11] Patel V Weobong B Weiss HA: The Healthy Activity Program (HAP), a lay Counsellor-Delivered Brief Psychological Treatment for Severe Depression, in Primary Care in India: A Randomised Controlled Trial. *Lancet.* 2017;389(10065):176–185. 10.1016/S0140-6736(16)31589-6 27988143 PMC5236064

[12] Poudyal A van Heerden A Hagaman A: Wearable Digital Sensors to Identify Risks of Postpartum Depression and Personalize Psychological Treatment for Adolescent Mothers: Protocol for a Mixed Methods Exploratory Study in Rural Nepal. *JMIR Res Protoc.* 2019;8(8):e14734. 10.2196/14734 31512581 PMC6746061

[13] Stewart CL Rashid Z Ranjan Y: RADAR-base: Major Depressive Disorder and Epilepsy Case.in UbiComp 2018. 2018 Association for Computing Machinery.: Singapore. 10.1145/3267305.3267540

[14] Passive data kit: Passive data kit. 2018; [cited 2020 14 April 2020] Reference Source

[15] Byanjankar P Poudyal A Kohrt BA: Supplementary File - StandStrong Counselor App. *Zenodo*.2020. 10.5281/zenodo.3709431

[16] Flow T: An end-to-end open source machine learning platform. n.d. Reference Source

[17] Kohrt BA Rai S Vilakazi K: Procedures to Select Digital Sensing Technologies for Passive Data Collection With Children and Their Caregivers: Qualitative Cultural Assessment in South Africa and Nepal. *JMIR Pediatr Parent.* 2019;2(1):e12366. 10.2196/12366 31518316 PMC6716492

[18] Maharjan SM Poudyal A van Heerden A: Passive Sensing Data Collection with Adolescent Mothers and Their Infants to Improve Mental Health Services in Low-Resource Settings: A Feasibility and Acceptability Study in Rural Nepal.2020. 10.21203/rs.3.rs-22755/v1

[19] Dmytro pbyanjankar vanheerdena: mmhss/sstrong-counselor: StandStrong v1.0 (Version v1.0). *Zenodo*.2020. 10.5281/zenodo.3709406

[20] pbyanjankar: mmhss/sstrong-import: First Release. *Zenodo*.2020. 10.5281/zenodo.3933064

[21] pbyanjankar: mmhss/sstrong-webhook: The first version of sstrong-webhook for Viber messaging service. *Zenodo*.2020. 10.5281/zenodo.3933074

[22] pbyanjankar: mmhss/sstrong-rest-server: The first version of sstrong-rest-server. *Zenodo*.2020. 10.5281/zenodo.3933119

