# Supplementary material for: Utilizing passive sensing data to provide personalized psychological care in low-resource settings
Source: Gates Open Res. 2021 Mar 2;4:118. Originally published 2020 Aug 5. [Version 2] doi: 10.12688/gatesopenres.13117.2 (PMC7940775; doi:10.12688/gatesopenres.13117.2)
Supplement: Peer review history

# Peer review: Reviewer response for version 2

**DOI:** 10.21956/gatesopenres.14457.r30457

**Published:** 2021-03-23

**Authors:** Shamsa Zafar

**Affiliations:** 1 Human Development Research Foundation, Rawalpindi, Pakistan; 2 Research Centre for Health and Wellbeing, Fazaia Medical College, Islamabad, Pakistan

I have no new comments.

Are the conclusions about the tool and its performance adequately supported by the findings presented in the article?

Yes

Is the rationale for developing the new software tool clearly explained?

Yes

Is the description of the software tool technically sound?

Yes

Are sufficient details of the code, methods and analysis (if applicable) provided to allow replication of the software development and its use by others?

Yes

Is sufficient information provided to allow interpretation of the expected output datasets and any results generated using the tool?

Yes

Reviewer Expertise:

Maternal mental health

I confirm that I have read this submission and believe that I have an appropriate level of expertise to confirm that it is of an acceptable scientific standard.

# Peer review: Reviewer response for version 2

**DOI:** 10.21956/gatesopenres.14457.r30458

**Published:** 2021-03-15

**Authors:** Randy P. Auerbach

**Affiliations:** 1 Columbia University, New York, NY, USA

Overall, the revised version is much improved. I have no further comments.

Are the conclusions about the tool and its performance adequately supported by the findings presented in the article?

Partly

Is the rationale for developing the new software tool clearly explained?

Yes

Is the description of the software tool technically sound?

Partly

Are sufficient details of the code, methods and analysis (if applicable) provided to allow replication of the software development and its use by others?

No

Is sufficient information provided to allow interpretation of the expected output datasets and any results generated using the tool?

Partly

Reviewer Expertise:

NA

I confirm that I have read this submission and believe that I have an appropriate level of expertise to confirm that it is of an acceptable scientific standard.

# Peer review: Reviewer response for version 2

**DOI:** 10.21956/gatesopenres.14457.r30115

**Published:** 2021-03-08

**Authors:** Olusola Ajilore

**Affiliations:** 1 Department of Psychiatry, University of Illinois at Chicago, Chicago, USA

This is a clearly-written manuscript detailing the development of the StandStrong platform which utilizes passive sensing data to optimize the delivery of therapy by paraprofessionals to a low resource population of teen mothers in Nepal. The authors do a good job of describing the platform and their rationale for design choices, equipment and selection of passive metrics. The use cases provide thorough real-world scenarios for the use of the StandStrong platform. 

 One issue that the authors should clarify is the battery usage by the StandStrong app which may be considerable since the app is recording data every 15 minutes. Given that this is being deployed in an under-resourced population, the platform draining the phone's battery power could severely impact successful implementation.

Are the conclusions about the tool and its performance adequately supported by the findings presented in the article?

Partly

Is the rationale for developing the new software tool clearly explained?

Yes

Is the description of the software tool technically sound?

Yes

Are sufficient details of the code, methods and analysis (if applicable) provided to allow replication of the software development and its use by others?

Yes

Is sufficient information provided to allow interpretation of the expected output datasets and any results generated using the tool?

Yes

Reviewer Expertise:

Digital mental health; Mood and anxiety disorders; Computational neuroimaging; Geriatric mood disorders

I confirm that I have read this submission and believe that I have an appropriate level of expertise to confirm that it is of an acceptable scientific standard.

# Peer review: Reviewer response for version 1

**DOI:** 10.21956/gatesopenres.14296.r29946

**Published:** 2020-12-16

**Authors:** Randy P. Auerbach

**Affiliations:** 1 Columbia University, New York, NY, USA

Overall, this is a very interesting project, and it has enormous potential for a high impact in low resource settings (as well as higher resourced settings). Below, I provide some issues the authors may wish to consider.

1. As written, it is not entirely clear how the passive sensor data is integrated into the clinical treatment. Perhaps the use cases can draw more attention to this issue, as I do believe this is where this project could really serve to help mothers in need.
2. The prospect of data visualization in real time is helpful. That said, it was not entirely clear how this would occur. I may be misreading, but it seems as though the data are "retrieved" once per week. Thus, how is it that data are then visualized on the phone in real time. Similarly, it is not evident how many of the features acquired will be used to direct treatment (e.g., audio clips). 
3. Broadly, I think it would be helpful to have more clear operationalization of the passive sensor indicators. Passive sensor research has now reached a stage in which these data are relatively easy to obtain but still post challenges in analyzing. Thus, it would be helpful to know how the authors intend to operationalize each variable and to some extent, if these variables are meant to reflect a specific construct (e.g., mother-child proximity as a proxy for attachment), it would be helpful to show some validation of these constructs. Before we intercede based on information culled from passive sensor data, I do believe these initial steps are critical.
4. Along similar lines, it is not evident how long data will be collected for in each subject. And, accordingly, for each variable, how will passive sensor data be analyzed to make use of the impressive temporal precision, which may capture important participant variability. 
5. Overall, I do believe there is enormous promise for this tool, and there is a clear need. I also believe that clearly defined constructs, as it relates to psychological/environmental constructs, will ensure that this research results in reproducible findings. 

Are the conclusions about the tool and its performance adequately supported by the findings presented in the article?

Partly

Is the rationale for developing the new software tool clearly explained?

Yes

Is the description of the software tool technically sound?

Partly

Are sufficient details of the code, methods and analysis (if applicable) provided to allow replication of the software development and its use by others?

No

Is sufficient information provided to allow interpretation of the expected output datasets and any results generated using the tool?

Partly

Reviewer Expertise:

Depression; Passive Sensor Data; Neuroimaging

I confirm that I have read this submission and believe that I have an appropriate level of expertise to confirm that it is of an acceptable scientific standard, however I have significant reservations, as outlined above.

# Response

**Published:** 2021-02-19

**Authors:** Anubhuti Poudyal

**Comment 1: As written, it is not entirely clear how the passive sensor data is integrated into the clinical treatment. Perhaps the use cases can draw more attention to this issue, as I do believe this is where this project could really serve to help mothers in need.**

 Response 1: Thank you so much for your comment. We have added the information on how passive sensor data was included in clinical treatment in Use Case 2.

*Following Case 1, the next step is to integrate the passive data in the counselor’s StandStrong app. The four types of passive data collected using smartphones and Bluetooth beacons can be visualized in the StandStrong app installed on the counselor’s tablet. The counselor receives daily passive data updates for each mother. Before the weekly psychosocial session, the counselors synchronize the latest data in their tablet to discuss during the counseling session.* *Once downloaded, the data is available offline for sessions which are typically held in mothers’ homes that might not have reliable internet access. The counselor then meets the mother in her scheduled weekly psychosocial counseling session.* *During the counseling session, the counselor shares and discusses the mother’s data with her using the StandStrong app on her tablet.* *This information is integrated as a part of the psychosocial session, particularly for behavior activation, that reinforces positive behaviors among the mothers.* *For example, they may show the proximity chart, GPS heat map, and activity tracking while discussing mothers’ daily life and behavioral patterns in the last one week. Any weekly change in behavioral patterns between sessions can also be discussed.* *Finally, counselors can provide suggestions to the mothers on behavior change for better recovery based on her passive data., with suggestions from counselors on what behaviors to adopt for better recovery. Besides interactions with the counselors during the psychosocial sessions* *, mothers can also send Viber text messages to counselors between sessions. These messages appear in the counselor’s StandStrong app on their tablet which they can respond through the app. Another reinforcing feature of StandStrong is “Awards” that are given to support certain self-care, daily routine, social interaction-related behavioral goals. This weekly progress is documented through passive data triggered behavioral change.*

**Comment 2: The prospect of data visualization in real time is helpful. That said, it was not entirely clear how this would occur. I may be misreading, but it seems as though the data are "retrieved" once per week. Thus, how is it that data are then visualized on the phone in real time. Similarly, it is not evident how many of the features acquired will be used to direct treatment (e.g., audio clips).**

 Response 2: Yes, the data is retrieved once per week and uploaded into the server for further processing. The counselor then syncs data into the StandStrong counselor app before sharing the data with the mother during weekly counseling sessions. So, we will not use the phrase “real-time”. However, since the data is available to the counselor between sessions, it gives access to patient information more frequently than a traditional counseling session. In most traditional counseling sessions, the between-session information is usually self-reported by the mothers, with no additional methods to monitor her behavior change. Successful collection of passive data from depressed mothers can help a counselor know mothers’ behaviors between sessions. Future implementation will look into getting this information in real-time. Features that were used in the app and during counseling sessions were – audio, proximity, activity, and GPS. We have added this information under Use Case 1.

*Once the data is analyzed, it is visualized in the StandStrong app. Four types of data are analyzed to be used in the counseling sessions – a) Audio data, b) Proximity, c) Activity and d) GPS data. *

1. *Audio –* *For the audio data, the audio recordings are not processed manually. The data file is directly uploaded to the TensorFlow app will compute the speech count percentage in the mother’s environment.* *An award was sent to the mother if there were presence of speech sounds during the day. *
2. *Proximity – Proximity chart shows hours per day that mother spent with her baby and hours spent apart. *
3. *Activity – Mothers’ activities are visualized in the app to show how much time she spends doing activities (running, walking, sitting etc.). *
4. *GPS – A heat map shows mothers’ geographical movement. *

**Comment 3: Broadly, I think it would be helpful to have more clear operationalization of the passive sensor indicators. Passive sensor research has now reached a stage in which these data are relatively easy to obtain but still post challenges in analyzing. Thus, it would be helpful to know how the authors intend to operationalize each variable and to some extent, if these variables are meant to reflect a specific construct (e.g., mother-child proximity as a proxy for attachment), it would be helpful to show some validation of these constructs. Before we intercede based on information culled from passive sensor data, I do believe these initial steps are critical.**

 Response 3: Thank you so much. We fully agree with the reviewer.  We have added a section “ ***Rationale for selected passive sensing domains***.” In this, we provide our rationale and potential overlap with psychological constructs. The added text is:

***Rationale for selected passive sensing domains.***

*We have included four domains that can create a picture of the daily life of the mothers. Mother-child proximity, along with audio data can capture mother-child interaction. Audio data combined with GPS is intended to eventually be a proxy for social support. Activity data will capture the physical activity of the mother as a proxy for physical health. The combined information from these passive data can be used to create a picture of the daily schedules of mothers. This can be a proxy for routinization and stability. An instability of daily schedule as determined from the passive data can be a proxy for stress. Although we are moving towards a clearer understanding of how to analyze and operationalize to maximize the benefit of mothers, the initial idea with this study was just to surface behaviors of interest. We wanted to see if it was possible to create an app and integrate passive data within the app and whether such information would inform the counselor during the session. We were mostly concerned if passive data, in general, was a feasible form of data that we could collect in a rural setting. With what we have learned and the advances in the field mean we can move beyond the exploratory, particularly in operationalizing the domains, and what integrating passive data into these behavioral patterns means for mothers.*

 We have also added the next steps in the Conclusion section-

*With what we have learned in this study and the current development in the field of passive sensing technology, we can move beyond this exploratory stage. We are continually updating our app and strengthening our methodology, especially in the operationalization of the passive sensor indicators. Future studies will focus on defining indicators and validating passive sensing data constructs.*

**Comment 4: Along similar lines, it is not evident how long data will be collected for in each subject. And, accordingly, for each variable, how will passive sensor data be analyzed to make use of the impressive temporal precision, which may capture important participant variability.**

 Response 4: The data was collected for the duration of the in-person intervention (5 weeks of the Healthy Activity Program’ plus one week prior and after the intervention; total duration = 7 weeks of passive sensing data for mothers in the intervention). Passive sensing data is supportive to psychosocial counseling. We have added the following information under “Output dataset”.

*Our analytic approach has been to quantize down to the 15-minute level and then generate 24-hour mappings that include missing data so as to be able to compare behavioral rhythms day by day and across sensors which may not always be collected simultaneously.*

**Comment 5: Overall, I do believe there is enormous promise for this tool, and there is a clear need. I also believe that clearly defined constructs, as it relates to psychological/environmental constructs, will ensure that this research results in reproducible findings.**

 Response 5: Thank you so much. We appreciate your feedback. As mentioned above, we have added a section entitled “ ***Rationale for selected passive sensing domains.***” This section provides a clearer psychological rationale for why we selected these particular domains in relation to psychological constructs. In addition, we are improving the app and we hope to implement stronger study designs to further define these constructs and obtain generalizable results that can lead to targeted enhancement of psychological interventions. 

# Peer review: Reviewer response for version 1

**DOI:** 10.21956/gatesopenres.14296.r30004

**Published:** 2020-11-30

**Authors:** Shamsa Zafar

**Affiliations:** 1 Human Development Research Foundation, Rawalpindi, Pakistan; 2 Research Centre for Health and Wellbeing, Fazaia Medical College, Islamabad, Pakistan

- The platform developed to collect data looks promising and can help women with depression.
- Since the platform developed is meant to help LMICs, the scalability might be an issue as it is the marginalized population who need to be trained on how to use Android mobile phones.
- The issue of privacy and confidentiality needs to be addressed. A study on the feasibility and acceptability of the tool with women from LMICs is needed to understand how women will feel about their information being shared with researchers and therapists. Do they feel that their personal space is being invaded and whether this could add anxiety? Acceptance of the family is also another area to be considered.

Are the conclusions about the tool and its performance adequately supported by the findings presented in the article?

Yes

Is the rationale for developing the new software tool clearly explained?

Yes

Is the description of the software tool technically sound?

Yes

Are sufficient details of the code, methods and analysis (if applicable) provided to allow replication of the software development and its use by others?

Yes

Is sufficient information provided to allow interpretation of the expected output datasets and any results generated using the tool?

Yes

Reviewer Expertise:

Maternal mental health

I confirm that I have read this submission and believe that I have an appropriate level of expertise to confirm that it is of an acceptable scientific standard, however I have significant reservations, as outlined above.

# Response

**Published:** 2021-02-19

**Authors:** Anubhuti Poudyal

**Comment 1: The platform developed to collect data looks promising and can help women with depression.**

 Response 1: Thank you so much for your feedback.

**Comment 2: Since the platform developed is meant to help LMICs, the scalability might be an issue as it is the marginalized population who need to be trained on how to use Android mobile phones.**

 Response 2: Thank you so much for your response. We have added information on scalability under Use Case 1. We have tried to highlight how we can best integrate passive technology into existing healthcare provisions in Nepal, particularly into government-implemented training for psychosocial counselors. We have added the following information to the manuscript -

*Psychosocial counselors can be trained to use the app. When procuring devices, low-cost Android smartphones like the Samsung J2 Ace phone are compatible to operate the StandStrong app. Thus, adding this passive sensing tool would be inexpensive from both technical and human resources perspectives. In terms of training, since it is passive data collection, mothers would not require training to use this technology. We will need to train the facilitators on using the technology, ensuring proper use during each visit, and integrating passive data in a psychosocial counseling session. Psychosocial counseling (HAP) is already a part of government-implemented training for psychosocial counselors in Nepal. Training of counselors in HAP in Nepal typically is delivered in approximately 5-days in the government curriculum. To integrate StandStrong, we recommend an extra day in the training. This is needed to train them on how to set up the passive sensing technology and troubleshooting. In addition, for each of the HAP sessions, the trainer should discuss with counselors what information is useful and can be incorporated into the session. Other training should include the use of awards and messaging with mothers. After training, counselors typically have weekly supervision with a HAP specialist, this will need to additionally include discussions of technological challenges and how the passive sensing information is being optimally used.*

**Comment 3: The issue of privacy and confidentiality needs to be addressed. A study on the feasibility and acceptability of the tool with women from LMICs is needed to understand how women will feel about their information being shared with researchers and therapists. Do they feel that their personal space is being invaded and whether this could add anxiety? Acceptance of the family is also another area to be considered.**

 Response 3: Thank you so much for your response. We have added information on privacy under Use Case 1.

*Prior to enrollment, project staff visits the mother to sign the consent form. Two layers of consent were sought. First, we obtained a written consent from the mothers to use the devices. Second, we explained the technology to the family members and only proceeded with the study after the family members gave verbal consent. We addressed any privacy and confidentially-related concerns from the participants and their family members. In our study, it was integral to seek participant as well as family consent to ensure we addressed any privacy or confidentiality concerns of the family. We also shared a one-page description of the study with the participant and family, so she could describe the study to her family and friends, even when the study team was not present. Some of the key considerations related to privacy are, a) educating participants on how to delete the files from the phones, b) ongoing interactions with the participant and family throughout the study duration to ensure easy communication, and c) ensuring good rapport building with the participants so they feel comfortable to raise privacy concerns (if any) throughout the study period. In future studies, we will explore processing the audio files in the phone itself, so we do not have to store the audio clips on the phones. We have discussed the issues related to privacy and confidentiality in detail in a separate paper.*
